# Characteristics of Attempted Suicide by Patients with Schizophrenia Compared with Those with Mood Disorders: A Case-Controlled Study in Northern Japan

**DOI:** 10.1371/journal.pone.0096272

**Published:** 2014-05-08

**Authors:** Takao Ishii, Eri Hashimoto, Wataru Ukai, Yohei Kakutani, Ryuji Sasaki, Toshikazu Saito

**Affiliations:** Department of Neuropsychiatry, School of Medicine, Sapporo Medical University, Sapporo, Japan.; The Nathan Kline Institute, United States of America

## Abstract

Recent reports suggest a lifetime suicide risk for schizophrenia patients of approximately 5%. This figure is significantly higher than the general population suicide risk consequently, detection of those at risk is clinically important. This study was undertaken to define the characteristics of suicide attempts by schizophrenia patients compared with attempts by patients with mood disorders. All patients were diagnosed using the ICD-10 criteria. The study population comprised 65 patients with F2 disorders (schizophrenia, schizotypal and delusional disorders), i.e., “the F2 group”, and 94 patients with F3 disorders (mood disorders), i.e., “the F3 group”, who presented in the clinical setting of consultation-liaison psychiatry. The F2 group had a significantly younger mean age and significantly higher ratios of ‘past/present psychiatric treatment’ and ‘more than 3 months interruption of psychiatric treatment’. In contrast, the ratios of ‘physical disorder comorbidity’, ‘alcohol intake at suicide attempt’ and ‘suicide note left behind’ were significantly higher in the F3 group. The F2 group attempted suicide by significantly more serious methods. Furthermore, ‘hallucination-delusion’ was the most prevalent motive in the F2 group and was the only factor that showed a significant association with the seriousness of the method of suicide attempt (OR = 3.36, 95% CI: 1.05–11.33).

## Introduction

Individuals with schizophrenia are at increased risk for suicide. The earlier consensus figure of a lifetime suicide risk for schizophrenia patients was 10%, which was based on reviews prior to 1990 [1], [2]. However, the recent authoritative review by Palmer et al. estimated a lifetime suicide risk of approximately 5% [3], a figure which remains significantly higher than the general population suicide risk. In addition, suicide attempts occur at a significantly greater rate in patients suffering schizophrenia than in the general population and are 5–10 times more common than completed suicide; it is estimated that 25–50% of schizophrenia patients will attempt suicide during their life time [4].

Detection of those at risk is clinically important because it has been suggested that prevention of suicide may be improved by more knowledge [5]. An earlier systematic review by Hawton et al. [6] in 2005 about risk factors for suicide in schizophrenia identified 29 high-quality data-containing studies which were analyzed for individual risk factors. In that review, they found that the important risk factors for suicide in schizophrenia were previous depressive disorders, previous suicide attempts, drug misuse, agitation or motor restlessness, fear of mental disintegration, poor adherence to treatment and recent loss [6]. Recently, Hor and Taylor [7] undertook systematic review of all original studies concerning suicide in schizophrenia published between June 2004 and January 2010 and identified 51 high-quality data-containing studies which were analyzed for individual risk factors. They found that risk factors with a strong association with later suicide included youth, male gender, and a high level of education. They also found that the number of prior suicide attempts, depressive symptoms, active hallucinations and delusions, and the presence of insight were illness related risk factors [7].

However, several reports have pointed out that risk prediction is imprecise and complex [7], [8]. Furthermore, recent reports suggest that the impact of known risk factors for suicide attempts by schizophrenics differs across ethnic groups [9], [10], although there have been recent efforts to standardize the nomenclature for the study of suicide and suicidal behaviors [11], [12].

In this study, we investigated schizophrenic patients with suicide attempts who presented in the clinical setting of consultation-liaison psychiatry. Initially, we worked on characterizing them in comparison with mood disorders, which have been most extensively investigated for their association with suicide and risk factors comparable to those in schizophrenia patients. Next, we sought to evaluate the factors influencing the seriousness of the method of attempted suicide.

## Methods

This case-controlled study was conducted at Sapporo Medical University Hospital in Sapporo, Japan. The subjects of the present study were selected from among 397 patients that had attempted suicide and consulted the department of Neuropsychiatry between December 2002 and July 2013. All patients were diagnosed using the ICD-10 criteria. Of them, 159 were extracted for the study population with F2 (schizophrenia, schizotypal and delusional disorders; 65 patients, i.e., “the F2 group”) or F3 (mood disorders; 94 patients, i.e., “the F3 group”). The F3 group included 11 patients diagnosed with bipolar affective disorder (F31): 1 with F31.0 (Bipolar affective disorder, current episode hypomanic), 8 with F31.3 (Bipolar affective disorder, current episode mild or moderate depression) or F31.5 (Bipolar affective disorder, current episode severe depression without psychotic symptoms), and 2 with F31.6 (Bipolar affective disorder, current episode mixed). The patients other than F31 in the F3 group are diagnosed with F32 (Depressive episode) or F33 (Recurrent depressive disorder), namely unipolar depression. The breakdown of 238 patients other than F2 and F3 group was as follows: 7 with F0 (organic, including symptomatic, mental disorders), 16 with F1 (mental and behavioral disorders due to psychoactive substance use), 13 with F4 (neurotic, stress-related and somatoform disorders), 1 with F5 (behavioral syndromes associated with physiological disturbances and physical factors), 70 with F6 (disorders of adult personality and behavior), 10 with F7 (mental retardation) or F8 (disorders of psychological development), 121 others.

Patients' medical records were reviewed and the following data extracted: age, gender, previous suicide attempt, within-1-year suicide reattempts, physical disorder, unemployed, requiring public assistance, living alone, past/present psychiatric treatment, more than 3 months interruption of psychiatric treatment, seriousness of the method of attempted suicide, alcohol intake at suicide attempt, method, motive and suicide note left behind.

Respecting the seriousness of the method of attempted suicide, the study population was classified into two groups according to the criteria shown in Table 1. These criteria are based on the classification of the suicide attempter by Asukai [13]. We modified it with two additions; 1 adding ‘or high-speed car’ to ‘jumping in front of a moving train’; 2 adding ‘Freezing’ to the methods since our hospital is located in the north part of Japan thus deliberate exposure to winter conditions can be a suicide method. Patients who used serious methods in their suicide attempts were classified as absolutely dangerous group (AD group) and those who used relatively mild methods as relatively dangerous group (RD group). Then we sought associations between the seriousness of the attempt method and particular characteristics of patient and suicide attempt other than ‘method’ in the F2 group by calculating the odds ratio (OR) and its 95% confidence interval for a 2×2 table between the seriousness of the attempt method and each factor. In this study, we did not use logistic regression analysis because the number of events per variable was relatively small. Peduzzi et al. pointed out that for events per variable (EPV) values of 10 or greater, no major problems occurred, and that for EPV values less than 10, however, the regression coefficients were biased in both positive and negative directions [14].

**Table 1:** Classification of the suicide attempt seriousness.

Methods	Absolutely dangerous group (AD group)	Relative dangerous group (RD group)
Jumping from a height	>10 m (From higher than fourth floor)	≦10 m (From lower than third floor)
Jumping in front of a moving train/high-speed cars	All cases AD group	-
Cutting/stabbing	Presence of the injury of internal organ	An internal organ does not have the injury
Drug overdosing	The fatal dose or more	Less than fatal dose A respirator, dialysis is unused
	Requiring a respirator or hemodialysis	
Poisoning	The fatal dose or more	Less than fatal dose A respirator, dialysis is unused
	Requiring a respirator or hemodialysis	
Hanging	All cases AD group	-
Burning	More than 30% of extent of burn class II	Other than left cases
	More than 30% of extent of burn class III	
	Smoke inhalation	
Drowning	Unconsciousness and respiratory failure are present at discovery	Other than left cases
Gassing	The thing which was coma at discovery	Other than left cases
Freezing	Unconsciousness and hypothermia at discovery	Other than left cases

The classification of the suicide attempter by Asukai (Psychiatry Clin Neurosci. 1995; 49: 91–97.) is partially modified.

Statistical processing was performed using SPSS 15.0 J for Windows. To compare investigational indicators in the F2 group and the F3 group, the t-test was used for the interval scale and chi-square or Fisher's exact test for the nominal scale. The significance level was set at 5% (two-tailed test), with significance probability shown by numerical figures.

All data were anonymously analyzed without individual patient consent due to the retrospective nature of the study. The Internal Review Board of Sapporo Medical University waived the need for individual informed consent and approved the study. Only demographic data of patients were obtained from the medical records and the data were recorded prior to analysis in a manner where subjects could not be identified either directly or through identifiers linked to the subjects.

## Results

Table 2 depicts the patient characteristics comparing the F2 and F3 groups. The ratios of ‘past/present psychiatric treatment’ and ‘more than 3 months interruption of psychiatric treatment’ were significantly higher in the patients with F2 disorders. In the patients with F3 disorders, the ratio of ‘physical disorder comorbidity’ was significantly higher than for the F2 group.

**Table 2:** Patient characteristics.

		Schizophrenic disorders group (the F2 group) N = 65	Mood disorders group (the F3 group) N = 95	p-value
age (years)	Mean (SD)	39.2 (14.7)	49.8 (16.9)	<0.001†
Gender	male (%)	40 (61.5)	44 (46.8)	n.s.‡
	female (%)	25 (38.5)	50 (53.2)	
Past suicide attempt history	yes (%)	21 (32.3)	20 (21.3)	n.s.‡
	no (%)	44 (67.7)	74 (78.7)	
	unkown (%)	0 (0)	0 (0)	
Within-1-year suicide reattempts	yes (%)	10 (15.4)	12 (12.8)	n.s.‡
	no (%)	55 (84.6)	82 (87.2)	
	unkown (%)	0 (0)	0 (0)	
Physical disorder	yes (%)	3 (4.6)	25 (26.6)	<0.001‡
	no (%)	61 (93.9)	67 (71.3)	
	unkown (%)	1 (1.5)	2 (2.1)	
Jobless	yes (%)	50 (76.9)	65 (69.1)	n.s.‡
	no (%)	11 (16.9)	22 (23.4)	
	unkown (%)	4 (6.2)	7 (7.4)	
Requirements for public assistance	yes (%)	14 (21.5)	10 (10.6)	n.s.‡
	no (%)	51 (78.5)	84 (89.4)	
	unkown (%)	0 (0)	0 (0)	
Living alone	yes (%)	14 (21.5)	24 (25.5)	n.s.‡
	no (%)	45 (69.2)	66 (70.2)	
	unkown (%)	6 (9.3)	4 (4.3)	
Past/present psychiatric treatment	yes (%)	54 (83.1)	54 (57.4)	0.01‡
	no (%)	11 (16.9)	40 (42.6)	
	unkown (%)	0 (0)	0 (0)	
More than 3 months interruption of psychiatric treatment	yes (%)	8 (12.3)	2 (2.1)	0.016§
	no (%)	57 (87.7)	92 (97.9)	
	unkown (%)	0 (0)	0 (0)	

† t-test;

‡ chi- square test;

§ Fisher's exact test.

Table 3 summarizes the characteristics of suicide attempts in group F2 compared with F3. Regarding ‘seriousness of the method of attempted suicide’, the ratio of individuals of AD group was significantly higher in the F2 group than the F3 group. The patients in the F2 group had significantly lower values for the ratio of ‘alcohol intake at suicide attempt’ and ‘suicide note left behind’. As for the method, there were significant differences; the ratio of individuals using ‘jumping from a height and jumping in front of a moving train or high-speed car’ was higher in the F2 group. Regarding the motive, there were also significant differences; the ratio of individuals with ‘hallucination-delusion’ was higher in the F2 group.

**Table 3:** Characteristics of suicide attempts.

		Schizophrenic disorders group (the F2 group) N = 65	Mood disorders group (the F3 group) N = 95	p-value
Seriousness of the suicide attempt method	Relatively dangerous (RD) (%)	17 (26.2)	50 (53.2)	<0.001‡
	Absolutely dangerous (AD) (%)	48 (73.8)	44 (46.8)	
Alcohol intake at suicide attempt	yes (%)	7 (10.8)	22 (23.4)	0.039‡
	no (%)	58 (89.2)	71 (75.5)	
	unkown (%)	0 (0)	1 (1.1)	
Method	Jumping from a height (%)	26 (40)	10 (10.6)	<0.001‡
	Jumping in front of a moving train/car (%)	10 (15.4)	1 (1.1)	
	Cutting/stabbing (%)	9 (13.8)	20 (21.3)	
	Medicinal drug overdosing (%)	9 (13.8)	28 (29.8)	
	Poisoning (%)	2 (3.1)	10 (10.6)	
	Hanging (%)	5 (7.7)	7 (7.4)	
	Burning (%)	2 (3.1)	6 (6.4)	
	Gassing (%)	1 (1.5)	10 (10.6)	
	Freezing (%)	0 (0)	4 (4.3)	
	Others (%)	2 (3.1)	3 (3.2)	
Motive	Hallucination-delusion (%)	46 (70.7)	8 (8.5)	<0.001‡
	Personal relationship (%)	8 (12.3)	28 (29.8)	
	Economic status (%)	1 (1.5)	14 (14.9)	
	Pain of illness (%)	4 (6.2)	21 (22.3)	
	Job/School issues (%)	3 (4.6)	9 (9.6)	
	Others (%)	2 (3.1)	5 (5.3)	
	Unkown/Obscure (%)	2 (3.1)	10 (10.6)	
A note left behind	yes (%)	0 (0)	8 (8.5)	0.021§
	no (%)	65 (100)	86 (91.5)	
	unkown (%)	0 (0)	0 (0)	

‡ chi- square test;

§ Fisher's exact test.

Finally, we examined the associations between the seriousness of the suicide attempt method and particular factors of patient characteristics and characteristics of suicide attempts other than ‘method’ in the F2 group by calculating the odds ratio (Table 4). Only ‘hallucination-delusion’ in Motive was identified as a significant factor (OR = 3.36, 95% CI: 1.05–11.33).

**Table 4:** Factors influencing the seriousness of suicide methods in the F2 group.

Characteristics	Seriousness of suicide methods		Odds Ratio (95% CI)
	AD group (N = 48)	RD group (N = 17)	
Gender (male)	30	10	0.95 (0.30–3.03)
Past suicide attempt history	17	4	1.60 (0.45–5.71)
Within-1-year suicide reattempts	9	1	3.38 (0.39–28.96)
Physical disorder	2	1	0.65 (0.55–7.71)
Jobless	37	13	1.07 (0.25–4.64)
Requirements for public assistance	9	5	0.50 (0.14–1.78)
Living alone	11	3	1.19 (0.28–5.40)
Past/present psychiatric treatment	40	14	0.95 (0.23–4.37)
More than 3 months interruption of psychiatric treatment	6	2	0.98 (0.18–4.02)
Alcohol intake at suicide attempt	6	1	2.09 (0.23–18.83)
(Motive)			
Hallucination-delusion	38	8	3.46 (1.05–11.33)
Personal relationship	5	3	0.49 (0.10–2.34)
Economic status	0	1	
Pain of illness	2	2	0.30 (0.04–2.31)
Job/School issues	3	0	
Others	2	0	
Unkown/Obscure	0	2	

AD group: Absolutely Dangerous group, RD group: Relative Dangerous group.

## Discussion

We collated the characteristics of suicide attempts in the F2 group (schizophrenic disorders) in comparison with the F3 group (mood disorders) in our university hospital between December 2002 and July 2013. Then we examined the factors influencing the seriousness of the method of suicide attempt by calculating odds ratios.

In this study, we demonstrated that the patients in the F2 group were significantly younger compared to the F3 groups. The onset age of both disorders may influence the result since the onset age of schizophrenic disorder is younger than that of mood disorder [15]–[17].

We found that the ratio of ‘physical disorder comorbidity’ was lower in the F2 group than in the F3 group. Physical illness has been shown to be strongly associated with suicide in schizophrenic patients [7]. However, it has also been postulated that there is a strong mutual relationship between mood disorders and physical disorders such as type 2 diabetes [18], [19], coronary heart disease [20], cancer [21], [22] and other chronic diseases.

Regarding psychiatric treatment, the ratio of ‘past/present psychiatric treatment’ and ‘more than 3 months interruption of psychiatric treatment’ were higher in the F2 group than in the F3 group. Several studies have reported a high risk of suicide in patients during the first episode of schizophrenia [23], [24]. In this study, 11 of 65 patients (16.9%) in the F2 group had not experienced any previous psychiatric treatments and all of them were first episode schizophrenics. However, the ratio of individuals who had not experienced any psychiatric treatments was significantly higher in the F3 group. The low psychiatry consultation background [25] in Japan may influence the overall results.

Several studies have pointed out that poor adherence to treatment is one of the risk factors for suicide attempts in schizophrenia patients [26], [27]. In this study, we defined poor adherence to treatment as ‘more than 3 months interruption of psychiatric treatment’. We found that this ratio of ‘more than 3 months interruption’ was significantly higher in the F2 group.

Regarding the methods of suicide attempt, individuals with schizophrenia tended to make attempts of moderate to extreme lethality [28], [29] or often died by violent means [30]. In the present study as well, the ratio of ‘absolutely dangerous’ methods was significantly higher in the F2 group, and patients in the F2 group tended to make suicide attempts by more serious methods such as ‘jumping from a height’ and ‘jumping in front of a moving train or high-speed car’.

Noteworthy was the finding that suicide attempts motivated by ‘hallucination-delusion’ were overwhelmingly more prevalent than those with other motives in the F2 group. Furthermore, ‘hallucination-delusion’ was the only factor that showed a significant association with the seriousness of the suicide attempt methods. In contrast, an earlier systematic review by Hawton et al. [6] in 2005 about risk factors for suicide in schizophrenia concluded that reduced risk was associated with hallucinations (OR = 0.50, 95% CI 0.35–0.71). However, a recent systematic review by Hor and Taylor [7] identified the association between the presence of positive symptoms, in particular auditory hallucinations and delusions, and an increased risk of suicide among patients with schizophrenia. While our study was consistent with this result, further studies are needed to confirm this because these differences may be due to the heterogeneity of the data.

The acute effects of alcohol use act as important risk factors for attempted or completed suicide among individuals with and without alcoholism [31], [32]. Acute alcohol use can precipitate suicidal behavior by inducing negative affect and impairing problem-solving skills, as well as aggravating impulsive personality traits [32], [33]. We found that the F2 group patients had significantly lower values for the ratio of ‘alcohol intake at suicide attempt’ in comparison with the F3 group. Interestingly, we also found that no patients in the F2 group left a suicide note behind while 8% of the patients in the F3 group left them behind. Little is known regarding suicide notes in schizophrenic patients. This result suggests that suicide in schizophrenic patients may tend to be impulsive rather than planned even if they do not use alcohol at the time of the attempt. Further studies are required to elucidate this.

The present study has several limitations that must be addressed. First, it was a retrospective study and, the number of cases was rather small since only one institute was involved. All the patients in the study were seen by psychiatrists in the consultation-liaison settings, the patients who completed suicide were not included. In this study, the F3 group was included the patient with bipolar disorders and unipolar depression. Grouping mood disorders as a whole may be an important limitation. We will consider investigating these issues in the future.

We investigated the clinical features of suicide attempts by schizophrenia patients and compared them with mood disorders, which have been most extensively investigated for association with suicide. The patients in the F2 group were significantly younger. The ratios of ‘past/present psychiatric treatment’ and ‘more than 3 months interruption of psychiatric treatment’ were significantly higher in the F2 group. Meanwhile, the ratios of ‘physical disorder comorbidity’, ‘alcohol intake at suicide attempt’ and ‘suicide note left behind’ were significantly higher in the F3 group. The patients in the F2 group were more likely to make suicide attempts using serious methods. Furthermore, ‘hallucination-delusion’ was the most prevalent motive and was the factor that showed a significant association with the seriousness of the method of attempted suicide. Therefore, psychiatrists and other medical staff should keep in mind that in patients with schizophrenia, anti-suicidal measures must be taken earlier than in patients with mood disorders. Particularly, managing the symptom of hallucination and delusion is very important to reduce the suicide risk.
